# Screening of Natural Antioxidants from Traditional Chinese Medicinal Plants Associated with Treatment of Rheumatic Disease

**DOI:** 10.3390/molecules15095988

**Published:** 2010-08-30

**Authors:** Ren-You Gan, Lei Kuang, Xiang-Rong Xu, Yuan Zhang, En-Qin Xia, Feng-Lin Song, Hua-Bin Li

**Affiliations:** 1 Guangdong Provincial Key Laboratory of Food, Nutrition and Health, School of Public Health, Sun Yat-Sen University, Guangzhou 510080, China; E-Mails: ganry_zsu@yahoo.cn (R.G.); kwung_1121@hotmail.com (L.K.); fly198013@yahoo.com.cn (Y.Z.); enqinxia@163.com (E.X.); sflin1986@163.com (F.S.); 2 Key Laboratory of Marine Bio-resources Sustainable Utilization, South China Sea Institute of Oceanology, Chinese Academy of Sciences, Guangzhou 510301, China; E-Mail: xuxr2000@yahoo.com (X.X.)

**Keywords:** medicinal plant, antioxidant capacity, total phenolic content

## Abstract

In order to find new sources of natural antioxidants, the antioxidant capacities of 50 medicinal plants associated with treatment of rheumatic diseases were systemically evaluated using the ferric-reducing antioxidant power (FRAP) and Trolox equivalent antioxidant capacity (TEAC) assays, and their total phenolic contents were measured by the Folin–Ciocalteu method. Their antioxidant activities of some of these plants were analyzed for the first time. The FRAP and TEAC assay results suggested that the antioxidant compounds in these plants possessed free radicals scavenging activity and oxidant reducing power. A positive linear correlation between antioxidant capacities and total phenolic contents implied that phenolic compounds in these plants could be the main components contributing to the observed activities. The results showed that *Geranium wilfordii*, *Loranthus parasiticus*, *Polygonum aviculare*, *Pyrrosia sheaeri*, *Sinomenium acutum* and *Tripterygium wilfordii* possessed the highest antioxidant capacities and total phenolic content among 50 plants tested, and could be rich potential sources of natural antioxidants.

## 1. Introduction 

Free radical-induced oxidative damage is involved in the pathogenesis of many chronic and degenerative diseases, such as cardiovascular disease, cancer, diabetes, neurodegenerative disease and ageing [1,2,3,4,5]. Reactive oxygen species (ROS), including superoxide free radical, hydrogen peroxide, hydroxyl free radical and singlet oxygen, play a key role in the oxidative damage of these diseases, which may result in DNA mutations, protein inactivation, lipid peroxidation, cell apoptosis or abnormal proliferation, eliciting the occurence of diseases from the cellular and molecular levels [6,7]. Antioxidants are substances capable of scavenging ROS and protecting from oxidative damage. Natural antioxidants, such as vitamins and polyphenols, have high antioxidant capacities and are abundant in many fruits and vegetables, whose consumption has been demonstrated to be inversely associated with the cardiovascular disease and some cancers [8]. 

The search for raw materials containing potent antioxidants continues to attract the attention of researchers. Fruit, vegetables, legume seeds and spices are all known to be rich sources of natural antioxidants, and medicinal plants are another important source for a wide variety of natural antioxidants. Recently there has been an increasing interest in natural antioxidants in Chinese medicinal plants (CMPs), and the health benefits of CMPs are thought to arise partly from the potential effects of their antioxidants on the reactive oxygen species produced in the human body. Evaluation of antioxidant activity of CMPs is very important because some plants possessing high antioxidant capacities, which are potentially valuable sources of natural antioxidants, could be screened out. In the literature, the antioxidant activities of many CMPs have been evaluated [9], and special attention has been paid to several types of CMPs, such as those possessing nutritious and tonic functions [10,11], anticancer activities [12], antiviral activities [13], heat-clearing properties [14], and blood circulation regulating actions [15,16]. Furthermore, several studies indicated that some CMPs possessed more potent antioxidant activities than common fruits and vegetables [12,17]. 

Chinese medicinal plants have been used for pharmaceutical and dietary therapy for thousands of years. In particular, there is a category of CMPs used to treat rheumatic disease, which is a series of connective tissue disorders, especially of the muscles and/or joints, pathologically characterized by inflammation, such as osteoarthritis, rheumatoid arthritis, systemic lupus erythematosus and ankylosing spondylitis. These medicinal plants have high anti-inflammatory abilities and can fight against the inflammation caused by rheumatic diseases. In addition, inflammation can be partly attributed to oxidative stress caused by ROS [18]. Furthermore, the anti-inflammatory activities of the medicinal plants could be from, at least in part, their antioxidant properties [19]. This prompted us to speculate that these medicinal plants, whose antioxidant activities were not yet evaluated systematically, could contain rich natural antioxidants. Thus, it is imperative to carry out a large-scale systematic screening of these CMPs to discover the plants with high antioxidant activities. 

The purpose of this study was to systematically evaluate the antioxidant capacities and total phenolic contents of 50 Chinese medicinal plants associated with the treatment of rheumatic diseases in order to find new potential sources of natural antioxidants, and to investigate the relationship between total phenolic contents and antioxidant capacities. The results from this study will be in useful to understand the antioxidant capacity profiles of these medicinal plants, and also helpful for investigation of antirheumatic drugs.

## 2. Results and Discussion 

### 2.1. Antioxidant capacities of 50 selected medicinal plants

The antioxidant capacities of 50 medicinal plants associated with the treatment of rheumatic diseases were evaluated using the ferric-reducing antioxidant power (FRAP) and Trolox equivalent antioxidant capacity (TEAC) assays, respectively. The FRAP assay estimates the ability to reduce ferric(III) ions to ferrous(II) ions, and the TEAC assay is used to determine the ability to scavenge ABTS^•+^ radicals, so both tests reflect the antioxidant capacities with different rationales and are simple, inexpensive, reproducible and commonly employed methods for evaluating antioxidant capacities [20,21,22].

The antioxidant capacities of these medicinal plants displayed a large variance in both the FRAP and TEAC assays (Table 1). For FRAP assay, the values ranged from 3.88 to 580.02 μmol Fe(II)/g dry weight of plant material, the median value was 90.05 μmol Fe(II)/g dry weight, the lower quartile and upper quartile of inter-quartile range were 42.08 and 159.39 μmol Fe(II)/g dry weight, respectively. Among these plants, the top ten plants with the highest antioxidant capacities were *Loranthus parasiticus*, *Geranium wilfordii*, *Pyrrosia sheaeri*, *Polygonum aviculare*, *Magnolia officinalis*, *Sinomenium acutum*, *Tripterygium wilfordii*, *Zanthoxylum nitidum*, *Chaenomeles speciosa* and *Erythrina variegate*, while *Poria cocos* was found to have the lowest antioxidant capacity. For the TEAC assay, the values ranged from 1.31 to 457.00 μmol Trolox/g dry weight, the median value was 45.25 μmol Trolox/g dry weight, the lower quartile and upper quartile of inter-quartile range were 32.38 and 104.20 μmol Trolox/g dry weight, respectively. Among the plants, the top ten with the highest antioxidant capacities were *Loranthus parasiticus*, *Geranium wilfordii*, *Magnolia officinalis*, *Tripterygium wilfordii*, *Polygonum aviculare*, *Pyrrosia sheaeri*, *Acanthopanax gracilistylus*, *Erythrina variegate*, *Sinomenium acutum* and *Pyrola calliantha*, and again *Poria cocos* was found to have the lowest antioxidant capacity. These medicinal plants had higher antioxidant capacities than common fruits, vegetables and microalgae [12,17,22], and could be potential rich sources of natural antioxidants.

**Figure 1:**
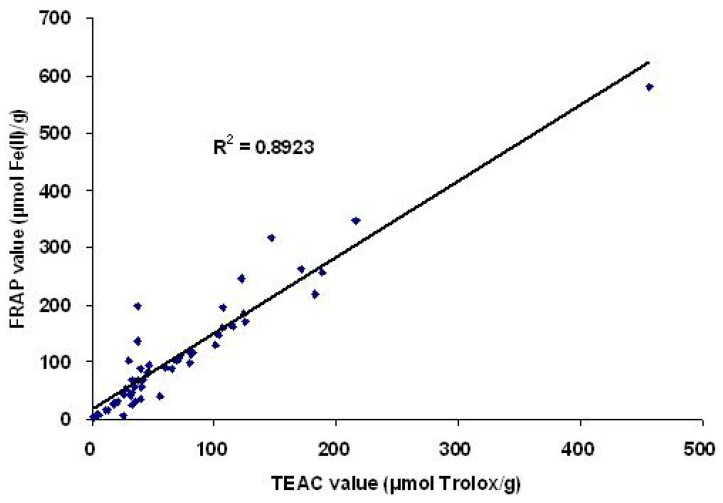
Correlation between the antioxidant capacities measured by the FRAP and TEAC assays.

A simple linear regression analysis was used to analyze the correlation between the FRAP values and TEAC values. The results exhibited a positive linear correlation (Figure 1). The regression equation Y = 1.3306 X + 16.725 was statistically significant (F = 397.485, *p* < 0.001), and the coefficient of determination R^2^ = 0.892, which suggested that the two methods were generally consistent for evaluating antioxidant capacities and antioxidants in these plants could not only reduce oxidants (ferric ions) but also scavenge free radicals (ABTS^•+^).

### 2.2. Total phenolic content of 50 selected medicinal plants

The Folin–Ciocalteu method was used to measure the total phenolic contents of these plants. It relies on the transfer of electrons from phenolic compounds to the Folin–Ciocalteu reagent in alkaline media [23], and is simple, reproducible and used in many studies [12,14,22,24]. Table 1 shows the total phenolic content of these medicinal plants and the values were extremely different, ranging from 0.10 to 29.67 mg GAE/g dry weight of plant material, the median value was 5.51 mg GAE/g dry weight, the lower quartile and upper quartile of inter-quartile range were 3.01 and 9.14 mg GAE/g dry weight, respectively. Among the 50 medicinal plants, the ten plants with the highest total phenolic contents were *Loranthus parasiticus*, *Pyrrosia sheaeri*, *Polygonum aviculare*, *Tripterygium wilfordii*, *Sinomenium acutum*, *Geranium wilfordii*, *Chaenomeles speciosa*, *Acanthopanax gracilistylus*, *Magnolia officinalis* and *Pyrola calliantha*. *Poria cocos* had the lowest total phenolic content.

### 2.3. Correlation between antioxidant capacities and total phenolic content

**Figure 2:**
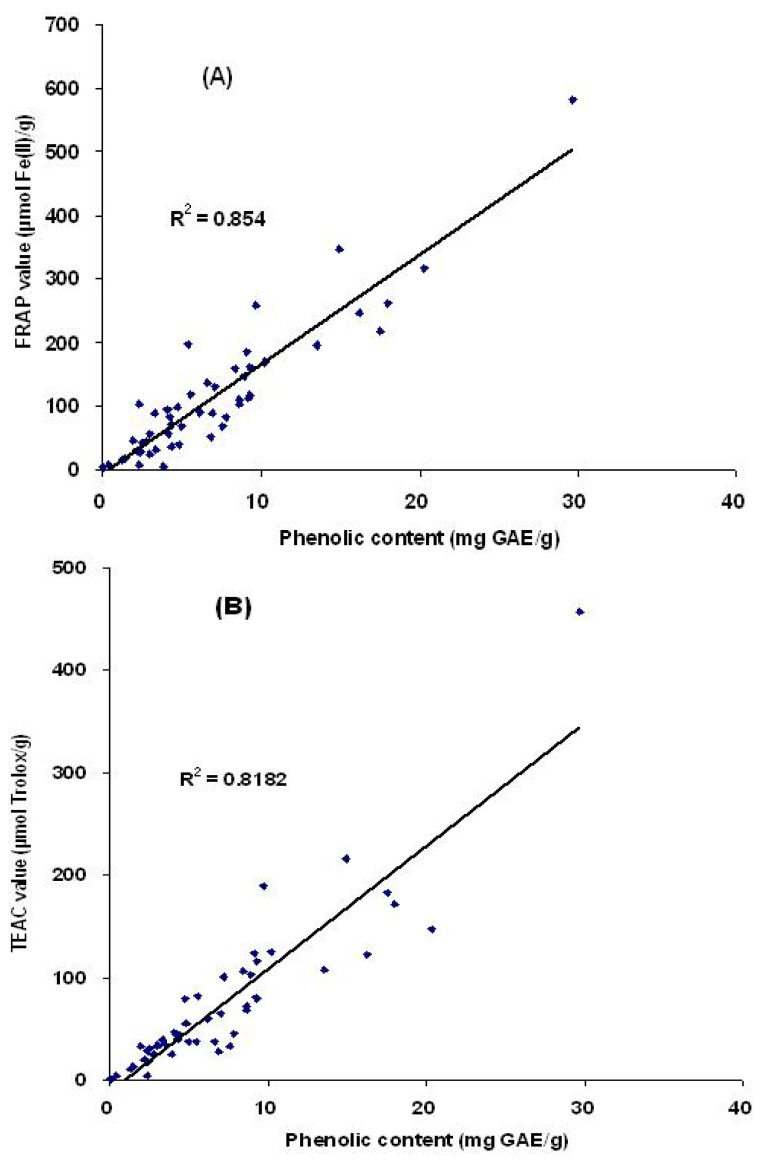
Correlation between the antioxidant capacities and total phenolic content. Antioxidant capacities were measured by the FRAP assay (A) and TEAC assay (B), respectively. GAE: gallic acid equivalents.

Simple linear regression analysis was used to analyze the correlation between the antioxidant capacities and the total phenolic content of 50 selected medicinal plants (Figure 2). For the correlation between FRAP values and total phenolic contents, the regression equation Y = 17.224 X – 6.7571 was statistically significant (F = 280.742, *p* < 0.001), and the coefficient of determination R^2^ was 0.854. 

For the correlation between TEAC values and total phenolic contents, the regression equation Y = 11.968 – 10.733 was also statistically significant (F = 216.107, *p* < 0.001), and the coefficient of determination R^2^ = 0.818. Figure 2 shows the positive linear correlation, and the total antioxidant capacities of these medicinal plants can be attributed to their phenolic compounds. In the literature, several other studies also found that phenolic compounds were the main contributors of the antioxidant capacities of the medicinal plants tested [21,25,26]. However, in some medicinal plants [27] it was also found that there was no significant linear correlation between the antioxidant capacities and the total phenolic contents, which suggested that other compounds, such as polysaccharides, might be the major antioxidant components in those plants [28].

## 3. Experimental

### 3.1. Chemicals and plant materials

2,4,6-Tri(2-pyridyl)-s-triazine (TPTZ), 6-hydroxy-2,5,7,8-tetramethylchromane-2-carboxylic acid (Trolox), 2,2′-azinobis(3-ethylbenothiazoline-6-sulfonic acid) diammonium salt (ABTS), Folin–Ciocalteu’s reagent and gallic acid were purchased from Sigma Aldrich (St. Louis, MO, USA). Iron (III) chloride 6-hydrate, iron (II) sulfate 7-hydrate, acetic acid, methanol, hydrochloric acid, acetic acid, sodium acetate, potassium persulphate and sodium carbonate were obtained from Tianjing Chemical Factory (Tianjing, China). All chemicals used in the experiment were of analytical grade. Fifty selected medicinal plants associated with treatment of rheumatic diseases were purchased from Beijing Tong-Ren-Tang drug retail outlet in Guangzhou, China. 

### 3.2. Sample preparation

The dry plant samples were ground to a fine powder with a special grinder for herbal medicine. A precisely weighed amount (0.5 g) of the powder was extracted with 10 ml of 80% methanol at 35 °C for 24 h in a shaking bath according to the literature [12]. All samples were then cooled down to the room temperature and centrifuged at 4,000 rpm for 10 min. The supernatant was recovered for the evaluation of antioxidant capacity and total phenolic content.

### 3.3. Ferric-reducing antioxidant power (FRAP) assay 

The FRAP assay was carried out according to the literature [20]. Briefly, the FRAP reagent was prepared from sodium acetate buffer (300 mM, pH 3.6), 10 mM TPTZ solution (40 mM HCl as solvent) and 20 mM iron (III) chloride solution in a volume ratio of 10:1:1, respectively. The FRAP reagent was prepared fresh daily and warmed to 37 °C in a water bath before use. One hundred microliters of the diluted sample was added to 3 mL of the FRAP reagent. The absorbance of the reaction mixture was then detected at 593 nm after 4 min in room temperature. The standard curve was constructed using FeSO_4_ solution, and the results were expressed as µmol Fe(II)/g dry weight of plant material. 

### 3.4. Trolox equivalent antioxidant capacity (TEAC) assay 

The TEAC assay was carried out to determine the free radical scavenging capacity using the ABTS^•+^ radical cation, according to the literature [29]. Briefly, the ABTS^•+^ stock solution was prepared from 7 mM ABTS^•+^ and 2.45 mM potassium persulfate in a volume ratio of 1:1, and then should be incubated in the dark for 16 h at room temperature and used within 2 days. The ABTS^•+^ working solution was prepared by dilution of the stock solution with ethanol to an absorbance of 0.70 ± 0.05 at 734 nm. All samples were diluted approximately to provide 20–80% inhibition of the blank absorbance. One hundred microliters of the diluted sample was mixed with 3.8 mL ABTS^•+^ working solution. The absorbance of the reaction mixture was then detected at 734 nm after 6 min of incubation at room temperature, and the percent of inhibition of absorbance at 734 nm was calculated. Trolox solution was used as a reference standard, and the results were expressed as µmol Trolox/g dry weight of plant material. 

### 3.5. Determination of total phenolic content 

The total phenolic contents were determined using Folin–Ciocalteu method according to the literature [23]. Briefly, diluted sample (0.50 mL) was added to 1:10 diluted Folin–Ciocalteu reagent (2.5 mL). After 4 min, saturated sodium carbonate solution (about 75 g/L, 2 mL) was added. After 2 h of incubation at room temperature, the absorbance of the reaction mixture was measured at 760 nm. Gallic acid was used as a reference standard, and the results were expressed as milligram gallic acid equivalent (mg GAE)/g dry weight of plant material. 

### 3.6. Statistical analysis

All the experiments were performed in triplicate, and the results were expressed as mean ± SD (standard deviation). The correlation between the antioxidant capacities and total phenolic contents was analyzed using the simple linear regression, and coefficient of determination (R^2^) was calculated. Statistical analysis was performed using SPSS 13.0 and MS Excel 2003. The significant difference was considered at *p* value ≤ 0.05.

## 4. Conclusions

The antioxidant capacities and total phenolic contents of 50 selected medicinal plants associated with the treatment of rheumatic diseases were evaluated using the FRAP and TEAC assays as well as the Folin-Ciocalteu method, respectively. Overall, these medicinal plants had relatively high antioxidant capacities and total phenolic contents. A significant correlation between the FRAP values and TEAC values suggested that antioxidants in these plants were capable of reducing oxidants and scavenging free radicals. A positive linear correlation between the antioxidant capacities and total phenolic contents indicated that the phenolic compounds could be the main contributors to the antioxidant capacities of these plants. Several plants (*Geranium wilfordii*, *Loranthus parasiticus*, *Polygonum aviculare*, *Pyrrosia sheaeri*, *Sinomenium acutum* and *Tripterygium wilfordii*) showed the highest antioxidant capacities and total phenolic contents among all the tested species, and they could be potential rich sources of natural antioxidants. Because of their strong antioxidant capacities, these plants are also potential in anti-inflammatory abilities. In the future, the specific compounds with high antioxidant capacities should be isolated, purified and identified from these plants to further develop natural antioxidants and investigate antirheumatic drugs. 

## Figures and Tables

**Table 1:** Antioxidant capacities and total phenolic contents of 50 medicinal plants.

Scientific Name	FRAP Assay (µmol Fe(II)/g)	TEAC Assay (μmol Trolox/g)	Phenolic Contents (mg GAE/g)
*Acanthopanax gracilistylus* W. W. Smith	170.19 ± 3.96	125.08 ± 7.32	10.23 ± 0.20
*Agadtacge rygisa* O. Kuntze.	95.19 ± 4.53	46.31 ± 2.39	4.10 ± 0.07
*Akebia trifoliata* (Thunb.) Koidz.	102.18 ± 4.32	29.15 ± 1.29	2.38 ± 0.11
*Alisma orientale* (Sam.) Juz.	5.54 ± 0.74	25.69 ± 2.11	3.90 ± 0.16
*Alpinia galanga* (L.) Willd.	82.21 ± 2.92	45.45 ± 0.82	4.25 ± 0.10
*Alpinia katsumadai* Hayat	42.89 ± 0.95	31.51 ± 0.99	2.52 ± 0.08
*Amomun kravanh* Pierre ex Gagnep	43.54 ± 2.73	25.50 ± 0.88	2.77 ± 0.14
*Amomum tsao-ko* Crevostet Lemarie	130.16 ± 2.85	100.61 ± 1.71	7.15 ± 0.17
*Amomum villosum* Lour	117.57 ± 1.43	80.16 ± 0.97	9.29 ± 0.13
*Angelica biserrata* Yuan et Shan	68.99 ± 3.26	32.67 ± 2.03	7.63 ± 0.58
*Artemisia capillaris* Thunb.	158.87 ± 7.50	106.55 ± 3.63	8.38 ± 0.20
*Atractylodes lancea* (Thunb.) DC.	28.15 ± 0.25	17.61 ± 2.56	2.41 ± 0.41
*Benincasa hispida* (Thunb.) Cogn.	57.28 ± 4.34	40.81 ± 0.42	4.21 ± 0.21
*Capsella bursapastoris* (L.) Medic.	69.99 ± 7.85	41.37 ± 4.01	4.35 ± 0.09
*Chaenomeles speciosa* (Sweet) Nakai	195.15 ± 2.78	107.61 ± 1.09	13.58 ± 0.13
*Clematis chinensis* Osbeck	82.27 ± 4.41	45.04 ± 0.70	7.85 ± 0.03
*Coix lacryma-jobi* L.	7.75 ± 0.15	4.69 ± 0.16	2.34 ± 0.44
*Cynanchum paniculatum* (Bge.) Kitag.	35.93 ± 0.62	39.45 ± 3.81	4.35 ± 0.10
*Dianthus superbus* L.	68.57 ± 2.00	37.12 ± 3.94	5.00 ± 0.07
*Dioscorea collettii* Hook. F.	16.05 ± 0.11	10.82 ± 0.28	1.31 ± 0.03
*Dioscorea nipponica* Makino.	39.64 ± 0.51	54.86 ± 3.27	4.82 ± 0.22
*Drosera burmannii* Vahl	99.80 ± 3.70	79.76 ± 4.18	4.76 ± 0.15
*Erythrina variegata* L.	185.91 ± 0.68	124.31 ± 5.69	9.12 ± 0.23
*Eupatorium fortunei* Turcz.	111.80 ± 9.46	71.93 ± 0.55	8.65 ± 0.10
*Gentiana macrophylla* Pall.	52.29 ± 1.69	27.55 ± 1.05	6.89 ± 0.48
*Geranium wilfordii Maxim.*	347.33 ± 7.99	215.98 ± 4.10	14.98 ± 0.64
*Homalomena occulta* (Lour.) Schott	32.30 ± 0.47	35.57 ± 0.61	3.40 ± 0.12
*Juncus effusus* L.	56.69 ± 7.33	34.77 ± 0.94	3.00 ± 0.18
*Kochia scparia* (L.) Schrad	103.22 ± 2.28	68.83 ± 1.69	8.63 ± 0.12
*Liquidambar formosana* Hance	118.44 ± 2.44	81.88 ± 11.11	5.58 ± 0.07
*Loranthus parasiticus* (L.) Merr.	580.02 ± 31.32	457.00 ± 6.41	29.67 ± 0.99
*Lysima chiachristinae* Hance	88.85 ± 1.44	65.30 ± 0.78	6.99 ± 0.05
*Magnolia officinalis* Rehd. et Wils.	257.45 ± 9.28	188.70 ± 12.01	9.68 ± 0.22
*Malva verticillata* L.	30.67 ± 2.17	20.32 ± 0.73	2.18 ± 0.07
*Morus alba* L.	46.96 ± 3.00	33.03 ± 2.38	1.96 ± 0.33
*Pinus tabulaeformis* Carr.	17.72 ± 0.25	13.30 ± 0.26	1.46 ± 0.01
*Piper kadsura* (Choisy) Ohwi	147.41 ± 3.64	103.41 ± 8.11	8.94 ± 0.16
*Plantago asiatica* L.	88.06 ± 13.26	39.94 ± 1.41	3.34 ± 0.43
*Plantago major* L.	137.23 ± 7.07	37.77 ± 0.85	6.62 ± 0.18
*Polygonum aviculare* L.	263.19 ± 4.73	171.65 ± 10.78	18.00 ± 0.25
*Polyporus umbellatus* (Pers) Fr.	7.93 ± 1.59	4.18 ± 0.09	0.38 ± 0.05
*Poria cocos* (Schw.) Wolf.	3.88 ± 0.15	1.31 ± 0.11	0.10 ± 0.01
*Pyrola calliantha* H. Andr.	160.96 ± 5.25	115.77 ± 10.03	9.31 ± 0.72
*Pyrrosia sheaeri* (Bak.) Ching	316.72 ± 4.82	147.26 ± 4.87	20.29 ± 0.17
*Siegesbeckia orientalis* L.	91.25 ± 4.39	60.23 ± 9.49	6.18 ± 0.14
*Sinomenium acutum* Rehd.et Wils.	245.94 ± 9.25	122.24 ± 1.74	16.21 ± 0.09
*Trachelospermum jasminoides* Lem.	113.22 ± 3.29	81.21 ± 6.10	9.19 ± 0.14
*Tripterygium wilfordii* Hook. F.	217.94 ± 2.49	181.98 ± 2.87	17.51 ± 0.30
*Vigna umbellata* Ohwi et Ohashi	24.74 ± 0.80	32.82 ± 1.62	3.01 ± 0.08
*Zanthoxylum nitidum* (Roxb.) DC.	198.25 ± 14.13	37.45 ± 1.95	5.44 ± 0.16
